# Supplementary material for: Divergent Driving Mechanisms Shape the Temporal Dynamics of Benthic Prokaryotic and Eukaryotic Microbial Communities in Coastal Subtidal Zones
Source: Microorganisms. 2025 Apr 30;13(5):1050. doi: 10.3390/microorganisms13051050 (PMC12114215; doi:10.3390/microorganisms13051050)
Supplement: Supplementary file 1 [file microorganisms-13-01050-s001.zip › microorganisms-3581439-supplementary.pdf]

Supporting Information

Divergent Driving Mechanisms Shape the Temporal Dynamics of Benthic Prokaryotic and Eukaryotic Microbial Communities in Coastal Subtidal Zones

Daode Ji [1,†], Jianfeng Zhang [1,†], Fan Li [2], Wensheng Li [3], Luping Bi [4,5], Wenlu Li [6,7], Yingjun Fu [6,7] and Yunfeng Wang [1,*]

[1] School of Ocean, Yantai University, Yantai 264005, China

[2] Shandong Marine Resource and Environment Research Institute, Shandong Provincial Key Laboratory of Restoration for Marine Ecology, Observation and Research Station of Laizhou Bay Marine Ecosystem, MNR, Yantai 264006, Shandong, PR China

[3] Laizhou Mingbo Aquatic Co., Ltd., Sanshan Island Street, Laizhou City, Yantai 261418, Shandong Province, China

[4] Fujian Province Key Laboratory for Coastal Ecology and Environmental Studies, College of the Environment and Ecology, Xiamen University, Xiamen 361102, China

[5] Key Laboratory of the Ministry of Education for Coastal and Wetland Ecosystem, College of the Environment and Ecology, Xiamen University, Xiamen 361102, China

[6] State Key Laboratory of Marine Environmental Science, College of Ocean and Earth Sciences, Institute of Marine Microbes and Ecospheres, Xiamen University, Xiamen 361102, China

[7] Fujian Key Laboratory of Marine Carbon Sequestration, Xiamen University, Xiamen 361102, China

† These authors contributed equally to this work.

* Corresponding author:
Yunfeng Wang, *E-mail:* yunfengwang1@126.com

Supplementary Figures

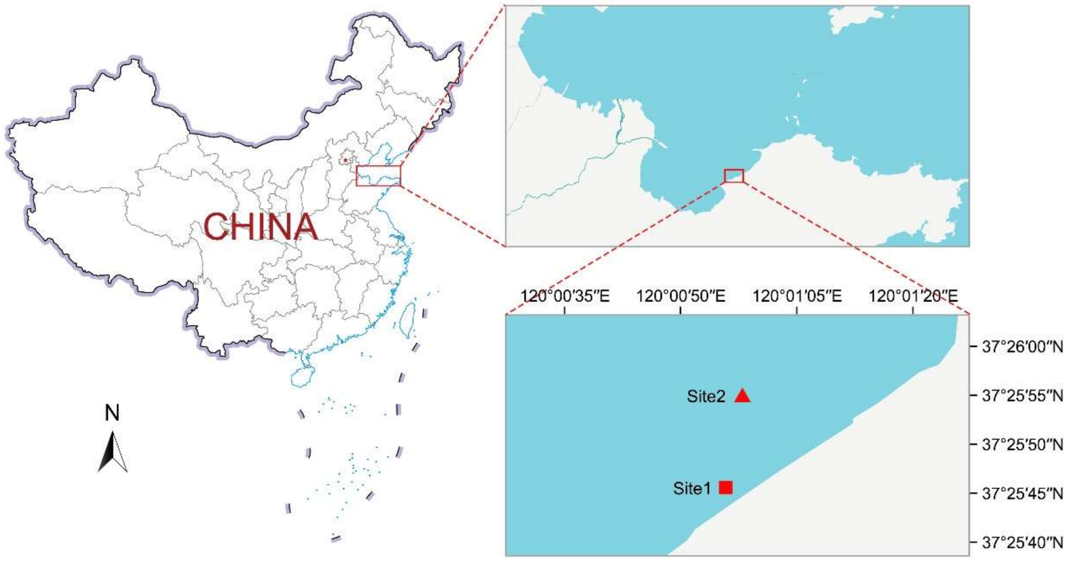

Figure S1. Location of the study area and sampling sites in the coastal subtidal zones of Sanshan Island.

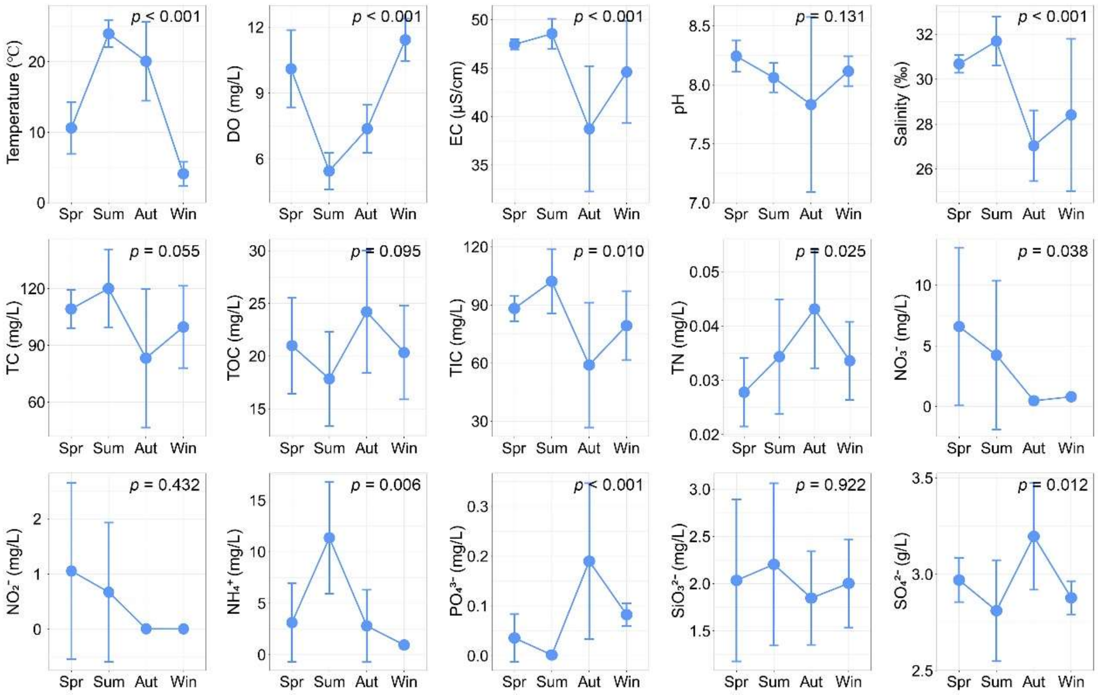

Figure S2. Temporal dynamics of 15 measured environmental factors in the coastal subtidal zones of Sanshan Island across different seasons. The significance of differences in environmental factors across different seasons was tested using the Kruskal-Wallis test.

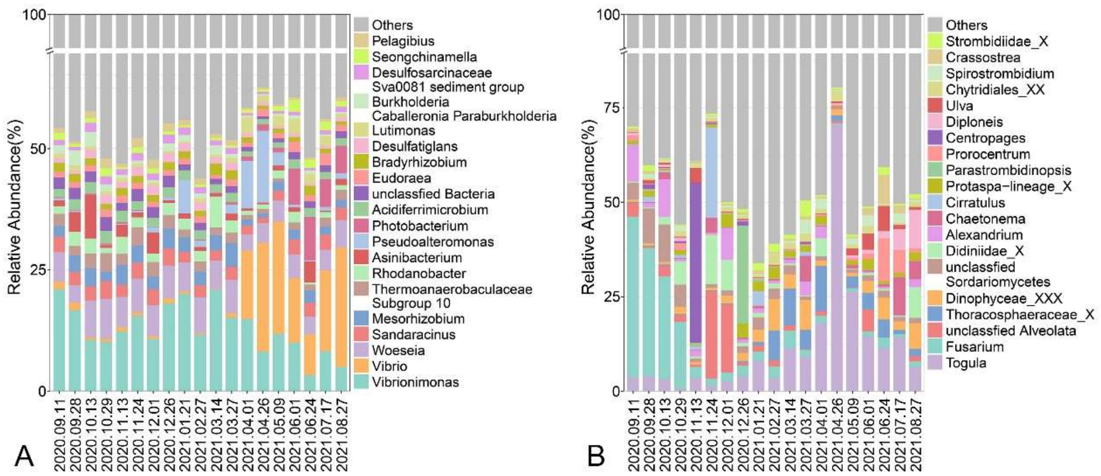

Figure S3. Temporal dynamics of relative abundance at the genus level (Top 20) in benthic prokaryotic (A) and eukaryotic (B) microbial communities from 11 September 2020 to 27 August 2021.

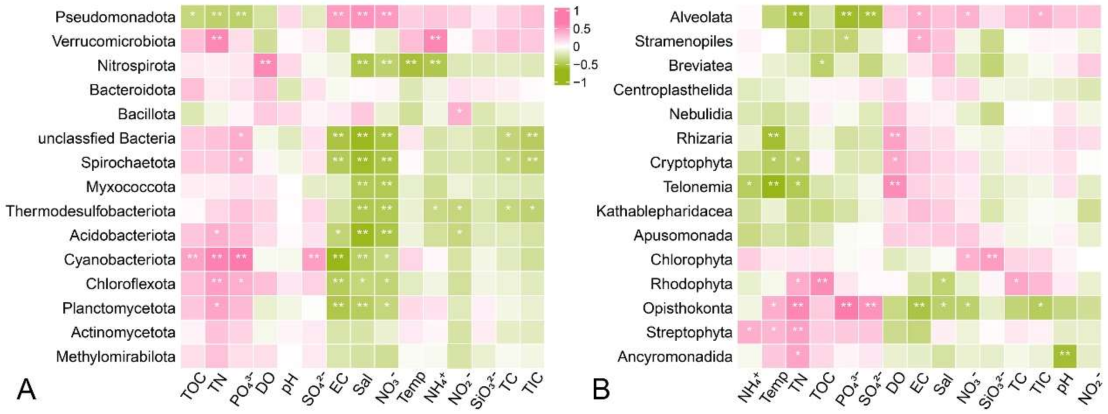

Figure S4. Heatmap of Pearson correlations between environmental parameters and dominant phyla (Top 15) in benthic prokaryotic (A) and eukaryotic (B) microbial communities. The color gradient in the heatmap represents Pearson correlation coefficients, with statistical significance indicated by p values. Significance levels are denoted as *$p < 0.05$; **$p < 0.01$; ***$p < 0.001$.

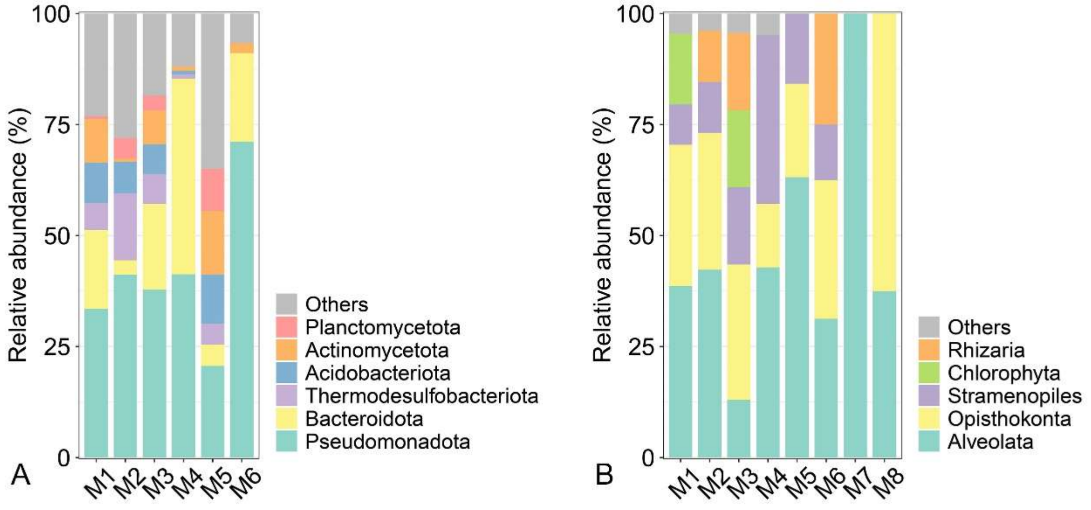

Figure S5. Taxonomic composition of the six largest modules at the phylum level in the benthic prokaryotic microbial co-occurrence network (A) and the eight largest modules at the phylum level in the benthic eukaryotic microbial co-occurrence network (B). Abbreviations: M, module.

Supplementary Tables

Table S1. Pairwise Adonis test comparisons of benthic prokaryotic and eukaryotic microbial communities across different seasons based on Bray-Curtis dissimilarity distance.

Microbial communities	Adonis test	
	R^2	*p*
Spring (Pro) vs. Summer (Pro)	0.194	0.001***
Spring (Pro) vs. Autumn (Pro)	0.234	0.001***
Spring (Pro) vs. Winter (Pro)	0.156	0.019*
Summer (Pro) vs. Autumn (Pro)	0.211	0.001***
Summer (Pro) vs. Winter (Pro)	0.262	0.001***
Autumn (Pro) vs. Winter (Pro)	0.130	0.020*
Spring (Euk) vs. Summer (Euk)	0.121	0.017*
Spring (Euk) vs. Autumn (Euk)	0.206	0.001***
Spring (Euk) vs. Winter (Euk)	0.137	0.003**
Summer (Euk) vs. Autumn (Euk)	0.222	0.001***
Summer (Euk) vs. Winter (Euk)	0.194	0.001***
Autumn (Euk) vs. Winter (Euk)	0.146	0.002**

Significance levels are denoted as *$p < 0.05$; **$p < 0.01$; ***$p < 0.001$. Abbreviations: Pro, prokaryotic microbial communities; Euk, eukaryotic microbial communities.

Table S2. Pearson correlations between environmental factors and alpha diversity indices.

	Pro		Euk	
	Shannon	Observed ASVs	Shannon	Observed ASVs
Temp	-0.09	-0.15	-0.35*	-0.48**
DO	0.11	0.21	0.30	0.46**
EC	-0.24	-0.24	0.53***	0.44**
pH	0.03	0.00	0.23	0.20
Sal	-0.38*	-0.44**	0.49**	0.28
TC	-0.03	-0.04	0.08	0.01
TOC	0.12	0.17	-0.38*	-0.32
TIC	-0.06	-0.07	0.16	0.08
TN	0.38*	0.37*	-0.43**	-0.41*
NO_3^-	-0.18	-0.30	0.11	0.08
NO_2^-	-0.33	-0.36*	0.09	-0.14
NH_4^+	-0.10	-0.21	-0.02	-0.16
PO_4^{3-}	0.16	0.22	-0.41*	-0.37*
SiO_3^{2-}	0.09	-0.02	-0.16	-0.14

The values represent Pearson correlation coefficients, with statistical significance indicated by *p* values. Significance levels are denoted as *$p < 0.05$; **$p < 0.01$; ***$p < 0.001$. Abbreviations: Pro, prokaryotic microbial communities; Euk, eukaryotic microbial communities.

Table S3. Topological properties of the benthic prokaryotic and eukaryotic microbial co-occurrence networks and their associated random networks.

Network	Nodes	Edges	AD	MD	CC	APL	GD	ND	PE	NE	r
Co-occurrence network (Pro)	710	5014	14.124	0.645	0.477	4.216	0.020	15.000	87.99%	12.01%	15.585
Random network (Pro)	710	5014	14.124	0.238	0.020	2.755	0.020	4.080			
Co-occurrence network (Euk)	452	202	4.475	0.734	0.447	5.407	0.022	13.000	94.03%	5.97%	13.798
Random network (Euk)	452	202	4.475	0.461	0.022	3.672	0.022	7.577			

Abbreviations: AD, average degree; MD, modularity; CC, clustering coefficient; APL, average shortest path length; GD, graph density; ND, network diameter; PE, positive edges; NE, negative edges; Pro, prokaryotic microbial communities; Euk, eukaryotic microbial communities. r = (CC/CC random)/(APL/APL random), r > 1 suggests ‘small-world’ properties, i.e., high efficiency and interconnectivity.

Table S4. The keystone taxa in the benthic prokaryotic and eukaryotic microbial co-occurrence networks.

ASV ID	Category	Network	Taxonomy (Domain; Kingdom; Phylum; Class; Order; Family; Genus)
ASV591	Network hubs	Pro	Bacteria; Pseudomonadota; Gammaproteobacteria; Enterobacterales; Moritellaceae; Moritella
ASV94	Module hubs	Pro	Bacteria; Pseudomonadota; Gammaproteobacteria; Steroidobacterales; Woeseiaceae; Woeseia
ASV214	Module hubs	Pro	Bacteria; Bacteroidota; Bacteroidia; Flavobacteriales; Flavobacteriaceae; Muriicola
ASV62	Module hubs	Pro	Bacteria; Pseudomonadota; Alphaproteobacteria; Kiloniellales; Kiloniellaceae; Pelagibius
ASV192	Module hubs	Pro	Bacteria; Pseudomonadota; Gammaproteobacteria; Burkholderiales; Nitrosomonadaceae; oc32
ASV188	Module hubs	Pro	Bacteria; Pseudomonadota; Alphaproteobacteria; Kiloniellales; Kiloniellaceae; Pelagibius
ASV119	Connectors	Pro	Bacteria; Spirochaetota; Leptospirae; Leptospirales; Leptospiraceae; Rs-H88 termite group
ASV25	Connectors	Pro	Bacteria; unclassfied Bacteria; unclassfied Bacteria; unclassfied Bacteria; unclassfied Bacteria; unclassfied Bacteria
ASV133	Connectors	Pro	Bacteria; Pseudomonadota; Gammaproteobacteria; Steroidobacterales; Woeseiaceae; Woeseia
ASV1409	Connectors	Pro	Bacteria; Bacteroidota; Bacteroidia; Chitinophagales; Saprospiraceae; Flavilitoribacter
ASV280	Connectors	Pro	Bacteria; Methylomirabilota; Methylomirabilia; Methylomirabilales; Methylomirabilaceae; unclassfied Methylomirabilaceae
ASV203	Connectors	Pro	Bacteria; Myxococcota; Polyangiia; Polyangiales; Sandaracinaceae; Sandaracinus
ASV27	Connectors	Pro	Bacteria; Bacteroidota; Bacteroidia; Flavobacteriales; Flavobacteriaceae; Actibacter
ASV32	Connectors	Pro	Bacteria; Pseudomonadota; Alphaproteobacteria; Rhodobacterales; Paracoccaceae; Nautella
ASV47	Connectors	Pro	Bacteria; Thermodesulfobacteriota; Desulfobulbia; Desulfobulbales; Desulfurivibrionaceae; MSBL7
ASV210	Connectors	Pro	Bacteria; Actinomycetota; Acidimicrobiia; Acidimicrobiales; Acidimicrobiaceae; Acidiferrimicrobium
ASV79	Connectors	Pro	Bacteria; Pseudomonadota; Gammaproteobacteria; Gammaproteobacteria Incertae Sedis; Unknown Family; Wenzhouxiangella
ASV141	Connectors	Pro	Bacteria; Actinomycetota; Rubrobacteria; Rubrobacterales; Rubrobacteriaceae; Rubrobacter
ASV1105	Connectors	Pro	Bacteria; Pseudomonadota; Gammaproteobacteria; Steroidobacterales; Woeseiaceae; Woeseia
ASV1225	Connectors	Pro	Bacteria; Acidobacteriota; Thermoanaerobaculia; Thermoanaerobaculales; Thermoanaerobaculaceae; Subgroup 1
ASV123	Connectors	Pro	Bacteria; Acidobacteriota; Vicinamibacteria; Vicinamibacterales; Vicinamibacteraceae; Luteitalea

ASV1263	Connectors	Pro	Bacteria; Patescibacteria; Saccharimonadia; Saccharimonadales; Saccharimonadaceae; Candidatus Saccharimonas
ASV1516	Connectors	Pro	Bacteria; Thermodesulfobacteriota; Desulfobacteria; Desulfobacterales; Desulfatibacillaceae; Candidatus Magnetananas
ASV2388	Connectors	Pro	Bacteria; Actinomycetota; Acidimicrobiia; Microtrichales; Microtrichaceae; Sva996 marine group
ASV250	Connectors	Pro	Bacteria; Actinomycetota; Acidimicrobiia; Actinomarinales; Actinomarinaceae; Candidatus Actinomarina
ASV901	Connectors	Pro	Bacteria; Verrucomicrobiota; Verrucomicrobiia; Verrucomicrobiales; Rubritaleaceae; Roseibacillus
ASV1745	Connectors	Pro	Bacteria; Actinomycetota; Thermoleophilia; Gaiellales; Gaiellaceae; Gaiella
ASV462	Connectors	Pro	Bacteria; Bacteroidota; Bacteroidia; Cytophagales; Cyclobacteriaceae; Fulvivirga
ASV671	Connectors	Pro	Bacteria; Bacillota; Bacilli; Bacillales; Bacillaceae; Bacillus
ASV85	Connectors	Pro	Bacteria; Pseudomonadota; Alphaproteobacteria; Rhodobacterales; Paracoccaceae; Ruegeria
ASV120	Connectors	Pro	Bacteria; Actinomycetota; Acidimicrobiia; Acidimicrobiales; Acidimicrobiaceae; Acidiferrimicrobium
ASV2597	Connectors	Pro	Bacteria; Bacillota; Clostridia; Lachnospirales; Lachnospiraceae; Oribacterium
ASV275	Connectors	Pro	Bacteria; Pseudomonadota; Gammaproteobacteria; Steroidobacterales; Woeseiaceae; Woeseia
ASV230	Connectors	Pro	Bacteria; Pseudomonadota; Alphaproteobacteria; Hyphomicrobiales; Methyloligellaceae; Methyloceanibacter
ASV495	Connectors	Pro	Bacteria; Bacteroidota; Bacteroidia; Chitinophagales; Saprospiraceae; Flavilitoribacter
ASV178	Connectors	Pro	Bacteria; Pseudomonadota; Gammaproteobacteria; Gammaproteobacteria Incertae Sedis; Unknown Family; Wenzhouxiangella
ASV304	Connectors	Pro	Bacteria; Pseudomonadota; Alphaproteobacteria; Rhodobacterales; Paracoccaceae; Sulfitobacter
ASV140	Connectors	Pro	Bacteria; Bacteroidota; Bacteroidia; Flavobacteriales; Flavobacteriaceae; Lutimonas
ASV638	Connectors	Pro	Bacteria; Bacteroidota; Bacteroidia; Flavobacteriales; Crocinitomicaceae; Salinirepens
ASV1323	Connectors	Pro	Bacteria; Acidobacteriota; Thermoanaerobaculia; Thermoanaerobaculales; Thermoanaerobaculaceae; Subgroup 1
ASV768	Connectors	Pro	Bacteria; Pseudomonadota; Alphaproteobacteria; Rhodobacterales; Paracoccaceae; unclassfied Paracoccaceae
ASV1182	Connectors	Pro	Bacteria; Pseudomonadota; Gammaproteobacteria; Arenicellales; Arenicellaceae; HTCC515
ASV1191	Connectors	Pro	Bacteria; Thermodesulfobacteriota; Desulfuromonadia; Desulfuromonadales; Geothermobacteraceae; Geothermobacter
ASV1415	Connectors	Pro	Bacteria; Thermodesulfobacteriota; Desulfobulbia; Desulfobulbales; Desulfurivibrionaceae; Desulfurivibrio
ASV548	Connectors	Pro	Bacteria; Pseudomonadota; Gammaproteobacteria; Pseudomonadales; Pseudohongiellaceae; Pseudohongiella
ASV27	Module hubs	Euk	Eukaryota; Alveolata; Dinophyceae; unclassfied Dinophyceae; unclassfied Dinophyceae; unclassfied Dinophyceae

ASV343	Module hubs	Euk	Eukaryota; Stramenopiles; Bacillariophyceae; Naviculales; Naviculaceae; Navicula
ASV129	Connectors	Euk	Eukaryota; Stramenopiles; Bacillariophyceae; unclassfied Bacillariophyceae; unclassfied Bacillariophyceae; unclassfied Bacillariophyceae
ASV159	Connectors	Euk	Eukaryota; Chlorophyta; Trebouxiophyceae; Chlorellales; Chlorellales_X; Picochlorum
ASV577	Connectors	Euk	Eukaryota; Opisthokonta; Nematoda; Enoplea; Enoplea_X; Enoplea_XX

Abbreviations: Pro, prokaryotic microbial communities; Euk, eukaryotic microbial communities.
